# Itch

**Published:** 1872-10

**Authors:** 


### ITCH

Is called scabies in medical books. It gives rise to an itching sensation, caused by a little animal constantly digging into the skin, feeding upon it, or preparing to deposit its eggs. By the intelligent use of the microscope, its size, form, and habits have been definitely determined. Its home is in the skin, just beneath the surface; there is the male and female; the latter lays the eggs, some fifty, in the course of its little life, and soon after dies. These are hatched within a week, and others soon begin to lay and hatch; multiplying with such rapidity as to spread all over the body, especially where the skin is most tender, as between the fingers. Shaking hands with a person having the itch is a frequent means of communicating it. It is said of General Washington, that on one occasion he was seen standing in the middle of the floor, greeting some common persons who came in, very cordially, but with his hands behind him; on surprise being expressed afterwards by a friend, he exclaimed, "They might give me the itch." It is not surprising that he should have been afraid, being a soldier, for it is found in armies everywhere, and so difficult is it to be got rid of, that it is an intolerable pest, sometimes for years; in fact, it is known as the seven years' itch, leaving the impression that it cannot be cured in less time; that it will run its course.

The itch insect is shaped like a turtle, the head nearly distinct from the body, with eight legs and four pair of jaws; there is a sucker at the end of each leg, somewhat in the shape of a bee's.

The same species of insect has been found in various animals, horses, lions, sheep, etc., circumstances making some difference in size and appearance.

The little animal runs over the skin until it finds a soft, tender point, then raises itself on its fore legs, begins to dig in with all its might, and ceases not until it has made a tunnel in a slanting direction, under the first layer of skin, where the eggs are deposited.

The skin of the human body grows like the finger-nails, which are pushed out; the skin on the surface is all the time wearing off, and the lower skin rises upwards, comes forward as it were to supply its place; consequently, the little eggs are constantly rising nearer the surface, and by the time they reach it, the new insect is hatched.

The more the skin is rubbed and scrubbed, the sooner it is worn off, especially if this is done with strong soap and warm water; this brings the eggs sooner to the surface than was intended; they get there before they are ready to be hatched; hence they are easily broken, and the insect is killed, or the strong alkali of the soap penetrates the egg-shell and destroys the life. Thus in a great many cases a free use of strong soap, warm water, and scrubbing, will cure the itch; in other words, scrupulous personal cleanliness.

But in some cases this is not efficacious, at least not so speedy a riddance as desired. If the ailing parts are plentifully rubbed with sweet or any other mild oil twice a day, the insect is smothered; hence all ointments used for that purpose have the one efficient ingredient, the oil. It will seldom fail to have a curative result to combine the soap and water washings and scrubbings, night and morning, followed by a good anointing; but sulphur ointment, made of lard and powdered brimstone, seems to have a peculiar efficiency, and may be used with confidence of a speedy and effectual riddance.

When attention has not been called to the case, as in children, prisoners, and neglected persons, the unfortunate insane especially, until the skin is full of scars, scabs, and scratches, with the correspondent inflammation, the bowels should be made to act freely once, if not twice a day, and the food eaten should be confined to coarse breads, fruits, and vegetables. It is not true that there is a seven years' itch, nor a year's itch, meaning thereby that it has such a time to run its course, and cannot be hurried any more than measles or small-pox; the means above named will cure, because after all it is a mechanical thing, the breaking of the eggs of the coming brood, and the killing of those already in existence. In all cases the inner clothing should be thoroughly washed and changed two or three times a week.

If there is any doubt in any case of the itching on the skin being the result of a living animal, take some of the scabs, put them in an ounce of water in which has been dissolved ten grains of caustic soda, bring it to a boil, let it settle, pour off the top, then examine this flaky settlement, when the skeletons of the itch insect, the acari, will be found.

There is a saying that "all signs fail in dry weather;" the principle applies to all diseases, sooner or later, for

#### MAN IS BORN TO DIE.

There are peculiarities in common itch, and in the habits and constitutions and bodily health of individuals, making a preparation which would cure ninety-nine in a hundred, entirely inefficient in the hundredth man; then again persons may be so situated as to be unable to obtain the best remedy or the constituents of the second best; hence a variety of

#### CURES FOR ITCH

are here appended, but in the use of any one of them observe strict personal cleanliness, especially of the skin; eat regularly, be in the open air, and avoid costiveness.

#### ITCH CURES.

1. Take one and one half ounce, that is, one table-spoonful, of spirits of camphor, and ten table-spoonfuls of the best vinegar, shake them well, and keep in a glass-stoppered bottle, or very tightly corked; rub some of this well into the skin with a coarse sponge; four or five good rubbings usually effect a cure of the common itch; if there is a good deal of irritation afterwards, bathe the parts in warm water.

2. Another cure, often effectual in twenty-four hours, is one part of sweet oil and two parts of storax, which is a balsam obtained by soaking an oriental bark in alcohol, then straining it; good drug stores have it on hand; simply smear the itching parts with it.

3. Common soft soap, rubbed so well into the skin as to make it smart and tingle pretty severely, repeating it well every day. If well done, a single application has cured. If a large part of the body is affected, cure a small portion at a time. In the course of an hour after the application, the soap may be washed off.

4. Take one ounce of bichromate of potash dissolved in six ounces of water. Rub it on the parts at once. A second application is seldom required; if so, let two or three days pass; as the mixture may make a sore. This same application has cured

RINGWORM, and quite a large number of skin affections.

5. Thirty drops of creosote, one ounce of lard, and thirty drops of strong vinegar, make an ointment to rub into the parts; it can be made stronger with creosote, if needed. Say two drachms of creosote to one ounce of common ointment, or hog's lard; if the weather is very warm, use simple cerate, instead of the lard. It must be rubbed on until it causes considerable smarting; this has been used with great success in curing

Barber's Itch.
Ecthyma.
Impetigo.
Pustules, of all sorts.
Ringworm.
Milk Scabs.
Common Itch.
Nail Tetters.
Chapped Hands, etc.

When tetter attacks the nails, put the ointment or a drop or two of the creosote itself between the nail and the skin or flesh.

When applied to pustules, put on the ointment and rub it in, or if the creosote is used, put it on gently, and when the pustule turns white, stop.

This same ointment can be used to advantage in softening the skin of the hands, when from any cause it has become hardened.

THE ITCH INSECT

will die in four hours in cold water; in three hours in sea water; in castor oil or sweet oil, in two hours; yet in Croton oil, which will in a short time fearfully blister the human skin, it lives four hours. It will die in lime water in forty minutes; in twenty minutes in strong vinegar; in spirits of turpentine, ten minutes; in the hydrodate of potash, in six minutes; in solution of arsenic, four minutes; in sulphuric acid, three minutes; in creosote, it dies instantly. But as it would be an interminable test to catch them and put them to these tests, the numerous methods of cure above named are given as the speediest methods of treatment and eradication.

6. Sometimes a cure is effected by simply sponging the parts well with vinegar, and then smearing them over with citrine ointment, obtained at any drug store.

7. But in one case every human remedy that had ever been known failed, ignominiously failed. A gentleman of means had tried to cure the itch for four years; it seemed to come on at intervals; at one time the intervals were six weeks, at others months, until it became perpetual, and there was danger of his

SCRATCHING HIMSELF TO DEATH.

On removing his shirt, his skin was a mass of scabs and scratches, and his garments all bloody; and for five months he had no comfortable rest, night or day. He was ordered to take a bath of water at ninety-five degrees, and remain in it half an hour; to this water was added two drachms of corrosive sublimate; there was to be enough water to cover his body up to his neck when lying down in the bath-tub. After the very first bath, he enjoyed a quiet night, the first in six months; on the next day there was a little itching, a second bath was taken at bedtime, and a third on the third night; at the end of six months the trouble had not returned.

Corrosive sublimate is a form of mercury, and had better be employed by the direction of a physician; and as soon as used, throw it away, as it is a violent poison when taken into the stomach in any form.

8. An ointment made of one ounce of chloroform, with half an ounce of hog's lard, has been often efficacious.

BENZOIN OINTMENT.

9. One ounce of the gum or resin, sixteen ounces of lard; put them in a tin or glass vessel, and immerse it in another vessel of hot water, stirring it from time to time in hot water for two hours; strain it without pressure, and stir it while cooling.

First have the whole body well washed with soap and warm water; wipe dry at bedtime, and rub the ointment well into every pin-point of the skin, being particular about all the creases and folds of the skin. One such rubbing will generally kill every insect in twenty-four hours; if not, repeat the ointment; but sometimes it requires twelve or fifteen days; but when it is remembered that it sometimes is an affliction which lasts for years, a pest and a disgust, it is recommended that one cure should be most effectually tried before another one is attempted.

10. Take flowers of sulphur or the very finest powdered brimstone, one ounce; hartshorn water, one ounce; hog's lard, eight ounces; make into an ointment, and after a good soap and warm water washing, rub some of it into the whole skin, night and morning, after each washing. Two or three applications generally cure, often one. Carbonate of ammonia is perhaps better than the water of ammonia or hartshorn.

